# Mechanisms and Regulation of Extracellular DNA Release and Its Biological Roles in Microbial Communities

**DOI:** 10.3389/fmicb.2017.01390

**Published:** 2017-07-26

**Authors:** Alejandra L. Ibáñez de Aldecoa, Olga Zafra, José E. González-Pastor

**Affiliations:** ^1^Laboratory of Molecular Adaptation, Department of Molecular Evolution, Centro de Astrobiología (Consejo Superior de Investigaciones Científicas/Instituto Nacional de Técnica Aeroespacial) Madrid, Spain; ^2^Experimental Sciences Faculty, Francisco de Vitoria University Madrid, Spain

**Keywords:** extracellular DNA, quorum sensing, competence, horizontal gene transfer, microbial communities, biofilms, social behavior

## Abstract

The capacity to release genetic material into the extracellular medium has been reported in cultures of numerous species of bacteria, archaea, and fungi, and also in the context of multicellular microbial communities such as biofilms. Moreover, extracellular DNA (eDNA) of microbial origin is widespread in natural aquatic and terrestrial environments. Different specific mechanisms are involved in eDNA release, such as autolysis and active secretion, as well as through its association with membrane vesicles. It is noteworthy that in microorganisms, in which DNA release has been studied in detail, the production of eDNA is coordinated by the population when it reaches a certain cell density, and is induced in a subpopulation in response to the accumulation of quorum sensing signals. Interestingly, in several bacteria there is also a relationship between eDNA release and the development of natural competence (the ability to take up DNA from the environment), which is also controlled by quorum sensing. Then, what is the biological function of eDNA? A common biological role has not been proposed, since different functions have been reported depending on the microorganism. However, it seems to be important in biofilm formation, can be used as a nutrient source, and could be involved in DNA damage repair and gene transfer. This review covers several aspects of eDNA research: (i) its occurrence and distribution in natural environments, (ii) the mechanisms and regulation of its release in cultured microorganisms, and (iii) its biological roles. In addition, we propose that eDNA release could be considered a social behavior, based on its quorum sensing-dependent regulation and on the described functions of eDNA in the context of microbial communities.

## Introduction

DNA molecules are not found exclusively within cells, but are an important component of the extracellular medium. Extracellular DNA (eDNA) has long been known as one of the most abundant molecules in slimy biological matrices formed by different microorganisms such as halophiles (Smithies and Gibbons, 1955; Catlin, 1956). It was subsequently detected in the supernatant of liquid cultures of numerous bacterial species, including *Neisseria meningitidis* (Catlin, 1960), *Bacillus subtilis* (Takahashi, 1962; Streips and Young, 1974), *Pseudomonas stutzeri*, and *Pseudomonas aeruginosa* (Hara and Ueda, 1981; Stewart et al., 1983), as well as many other species (Lorenz and Wackernagel, 1994). Moreover, eDNA has been revealed as an important component of the extracellular matrix of multicellular communities such as the biofilms formed by bacteria, archaea, and fungi (Chimileski et al., 2014b; Okshevsky and Meyer, 2015).

Furthermore, the phenomenon of eDNA release is not only observed under laboratory conditions, but eDNA is widespread in natural environments and can be found in most samples from aquatic and terrestrial ecosystems colonized by microorganisms (Paul et al., 1987; Tani and Nasu, 2010). In those ecosystems, eDNA may originate in part by the lysis of microbial cells due to lytic phages or necrosis, or by specific mechanisms that have been described in cultivable microorganisms, as summarized below, such as autolysis and active secretion systems, as well as through its association with extracellular membrane vesicles. The term environmental DNA, which is also abbreviated as eDNA in the literature (Taberlet et al., 2012), should not be confused with that of extracellular DNA. Environmental DNA refers to the total DNA that can be extracted from an environmental sample, which is a complex mixture of cellular genomic DNA from living organisms and extracellular DNA.

Microorganisms employ intercellular communication within large groups of cells to coordinate different processes, such as bioluminescence, antibiotic production, sporulation, competence, swarming motility, and the formation of biofilms and fruiting bodies. Thus, an individual cell activates specific functions by detecting the presence of a critical population density, and the whole community behaves as a multicellular organism (Shapiro, 1998; Waters and Bassler, 2005; Camilli and Bassler, 2006; González-Pastor, 2012). Interestingly, most of the known mechanisms of eDNA release are regulated by quorum sensing (QS): a cell density-dependent communication system that regulates cooperative behaviors. Therefore, eDNA is usually produced in response to an increase in the cell density of the population. In addition, it is noteworthy that in several bacteria the eDNA release pathways are related to the development of natural competence, which enables the cells to be transformed by DNA.

This review aims to provide a general perspective on eDNA research, summarizing the studies about its presence in the environment, the mechanisms and regulation of its release as described in various cultured microorganisms, and the different biological roles proposed for eDNA, such as biofilm formation, DNA damage repair, horizontal gene transfer (HGT) and its use as a source of nutrients. Moreover, we propose that eDNA release could be considered a social behavior since, in most of the microorganisms studied, it is the result of a coordinated response of the cells within the population and also that eDNA is present and is an important compound in microbial communities.

## eDNA is widespread in the environment

eDNA has been detected in a wide range of environments such as marine and freshwater ecosystems, sediments, soils, and biofilms, and it has been shown to be derived from bacteria, archaea, eukaryotes, and viruses. Thus, the presence of eDNA is vastly widespread making it a more important and common phenomenon feature than previously considered. Although eDNA in the environment has now been better characterized, further studies are needed to understand its role in maintaining ecosystems maintenance and, more broadly, in evolution.

### eDNA in aquatic environments

In marine, oceanic and freshwater ecosystems, the term “dissolved DNA” is usually used to refer to the whole amount of DNA that can be extracted from water samples. eDNA concentrations have been reported ranging from 0.03 to 88 μg L^−1^ (Deflaun et al., 1986; Karl and Bailiff, 1989; Nielsen et al., 2007), a wide range as a result of the physico-chemical, environmental and geographical difference. Quantities of eDNA in various aquatic environments and sediments have been updated by Torti et al. (2015).

In marine environments, the minimum DNA concentration has been reported to be <1 μg L^−1^ in oligotrophic oceans (Paul et al., 1987), while it may reach a maximum of 44 μg L^−1^ in subtropical estuaries (Deflaun et al., 1986). In general, the concentration of eDNA decreases with increasing distance to the coast and with depth (Deflaun et al., 1986; Karl and Bailiff, 1989).

On the other hand, Deflaun et al. (1986) studied freshwater environments and reported concentrations ranging from 1.74 to 7.7 μg L^−1^. In this study the amount of eDNA in eutrophic freshwater areas was found to be similar to those obtained for marine offshore ecosystems (Deflaun et al., 1986).

Interestingly, eDNA can be incorporated into other bacteria as demonstrated by the use of [^3^H] thymidine labeling (Paul et al., 1987). The eDNA may play different roles in the ecosystem, for instance, Pillai and Ganguly (1972) reported for the first time that it forms part of most dissolved organic matter (DOM) in marine ecosystems. Since then, studies on eDNA turnover rates provided a variety of results ranging from hours to months (Nielsen et al., 2007). Another remarkable result is that the size of eDNA molecules (low or high molecular weight) has been shown to affect the diversity of cultured microorganisms from a marine environment (Lennon, 2007), which is described in detail below, in the section “eDNA function as a source of nutrients.” In oligotrophic freshwaters and eutrophic areas the turnover rates are considerably faster than in marine and oceanic ecosystems (9.62 ± 3, 6 h and 10.5 ± 2, 1 h, respectively; Paul et al., 1989). Besides, studies using plasmidic DNA showed that DNA degrades more rapidly at the surface than at depth (Nielsen et al., 2007).

### eDNA in sediments and soil

According to Dell'Anno and Corinaldesi (2004) and Dell'Anno and Danovaro (2005), deep-sea marine sediments are the largest reservoir of DNA in the world oceans, a total of 0.50 ± 0.22 Gt of DNA within the 10 cm surface. Basically, they found that the concentration of eDNA in sediments was 4.3 times higher than the DNA associated with the entire bacterial community, and 2–3 times higher than the concentration in water (Paul et al., 1989). In this study, it was also calculated that this DNA provides 4% of carbon, 7% of nitrogen, and 47% of phosphate required by prokaryotes per day, which implies its important role as a source of organic matter for microbial communities (Dell'Anno and Corinaldesi, 2004; Dell'Anno and Danovaro, 2005).

With respect to eDNA in soils, reports vary depending on the type of sample. A maximum of 1,950 ng g^−1^ has been measured (Niemeyer and Gessler, 2002), however it is more usual to find concentrations of ~80 μg g^−1^ (Ogram et al., 1987; Selenska and Klingmüller, 1992; Nielsen et al., 2007).

It has been shown that eDNA is highly stable both in sediments and in soil, thereby being preserved for long periods of time. For instance, eDNA has been found in sediment samples as old as 450,000–800,000 years (DeSalle et al., 1992; Nielsen et al., 2006; Willerslev et al., 2007; Corinaldesi et al., 2008). Thus, turnover rates are long (ranging from 29 to 93 days) when compared with estimates in aquatic environments (Dell'Anno and Corinaldesi, 2004). This slower degradation (Novitsky, 1986), was found in sediments of different ages (as old as 10,000 years) and environmental conditions (Corinaldesi et al., 2008); and it is thought that is the result of its adsorption to sediment or soil particles. Although increased DNAase activity has been observed in sediments, probably due to higher amounts of DNA (Corinaldesi et al., 2008; Lu et al., 2010), other experiments have shown that by adsorption, DNA is protected against hydrolysis and DNAases (Paul et al., 1989; Corinaldesi et al., 2008). However, it is noteworthy that DNA adsorption is clearly influenced by the type of sediment (Greaves and Wilson, 1970; Ogram et al., 1987). Interestingly, transformation rates were assessed to be faster in sediments than in the water column (Paul et al., 1989). Moreover, soil experiments demonstrated that the transformation rate is not affected by adsorption, desorption or binding processes, nor by cell lysates present after cell death. In contrast, factors such as the type of sediment and its pH influence the process (Khanna and Stotzky, 1992; Lee and Stotzky, 1999; Gallori et al., 1994). Taken together, these data support the idea that sediments and soil particles are enhancing HGT by preserving DNA and facilitating the transformation of naturally competent microorganisms.

### Sources of extracellular DNA in the environment

eDNA in the environment may derive from: (i) active release from physiologically active cells, (ii) passive release from dead cells, and (iii) viruses. As described below, most bacteria and archaea studied to date are able to produce eDNA. Passive release of DNA occurs as a result of cell death caused by viral infection, antimicrobial agents, predation, etc. However, the origin of eDNA in the environment is poorly characterized. Even if some specific ecosystems have been deeply studied, such as hypersaline Mediterranean basins (DHAB) where 85% of the DNA has been shown to be derived from prokaryotic cell death (Corinaldesi et al., 2014), further studies are needed to accurately describe the specific origin of eDNA in different environments. Nonetheless, some interesting studies have shed light on that issue. For instance in both aquatic environments and sediments it has been demonstrated that 50% of the bulk eDNA is free DNA, while the rest is associated with viral particles or colloids. Within the bounded fraction, 17–30% is considered of viral origin, while the rest is of bacterial and eukaryotic origin (20–33%; Jiang and Paul, 1995; Nielsen et al., 2007). These proportions vary when referring to soil samples, where DNA origin is mainly fungal, but it also includes DNA from plants (released by decomposition, mechanical disruption, and pathogen-induced degeneration) and from bacteria and archaea (Nielsen et al., 2007).

## eDNA production in cultured microorganisms

As aforementioned, many microorganisms are able to release eDNA by different mechanisms (Table 1). Some of the most studied and interesting examples of eDNA production in cultured bacteria are reviewed below.

**Table 1:** Microorganisms producing eDNA and their release mechanisms.

**Microorganism**	**Mechanism of eDNA release**	**Regulation of eDNA release**	**Function of eDNA**	**References**
**GRAM-NEGATIVE BACTERIA**				
*Acinetobacter calcoaceticus*	Lysis	Unknown	Unknown	Palmen and Hellingwerf, 1995
*Campylobacter jejuni*	Autolysis	Unknown	Biofilm matrix	Svensson et al., 2014
*Caulobacter crescentus*	Lysis	Unknown	Biofilm dispersal	Berne et al., 2010
*Haemophilus influenzae*	Unknown	Unknown	Biofilm matrix	Izano et al., 2009
*Helicobacter pylori*	Vesicles	Unknown	Biofilm matrix	Grande et al., 2011, 2015
*Neisseria gonorrhoeae*	Type IV secretion system	Unknown	HGT and biofilm	Hamilton et al., 2005; Steichen et al., 2011
*Neisseria meningitidis*	Lysis	Unknown	Biofilm matrix	Lappann et al., 2010
*Pseudomonas aeruginosa*	Vesicles/prophage	QS	Biofilm matrix and nutrient source	Kadurugamuwa and Beveridge, 1996; Allesen-Holm et al., 2006; Mulcahy et al., 2010
*Pseudomonas chlororaphis*	Autolysis	Unknown	Biofilm matrix	Wang et al., 2016
*Pseudomonas fluorescens*	Unknown	Unknown	Unknown	Catlin and Cunningham, 1958
*Pseudomonas stuzeri*	Lysis	Unknown	HGT	Stewart et al., 1983
*Rhodovulum sulfidophilum*	Unknown	QS	Biofilm matrix (flocculation)	Watanabe et al., 1998 Suzuki et al., 2009
*Shewanella oneidensis*	Autolysis	Phage-induced upon iron-mediated oxidative stress	Biofilm matrix and nutrient source	Gödeke et al., 2011a,b; Binnenkade et al., 2014
*Vibrio costicolus*	Unknown	Unknown	Unknown	Smithies and Gibbons, 1955
*Xanthomonas citri*	Unknown	Unknown	Biofilm matrix	Sena-Vélez et al., 2016
**GRAM-POSITIVE BACTERIA**				
*Bacillus cereus*	Unknown	Unknown	Biofilm matrix	Vilain et al., 2009
*Bacillus subtilis*	Lytic-independent mechanism	QS (early competence)	HGT, nutrient source?	Takahashi, 1962 Zafra et al., 2012
*Deinococcus radiodurans*	Unknown	Unknown	DNA repair	Boling and Setlow, 1966
*Enterococcus faecalis*	Fratricidal-mechanism of autolysis	Unknown	Biofilm matrix	Thomas et al., 2008, 2009
*Lysteria monocytogenes*	Unknown	Unknown	Biofilm matrix	Harmsen et al., 2010
*Micrococcus halodenitrificans*	Unknown	Unknown	Unknown	Smithies and Gibbons, 1955
*Micrococcus sodonensis*	Unknown	Unknown	Unknown	Campbell et al., 1961
*Mycobacterium avium*	Unknown	Unknown	Biofilm matrix	Rose et al., 2015
*Staphylococcus aureus*	Autolysis	QS	Biofilm matrix	Rice et al., 2007 Brackman et al., 2016
*Staphylococcus epidermidis*	Autolysis	Unknown	Biofilm matrix	Qin et al., 2007
*Staphylococcus lugdunensis*	Lytic-independent mechanism	Competence (*comEB)*	Biofilm matrix	Rajendran et al., 2015
*Streptococcus gordonii*	Lytic-independent mechanism	QS (competence)	Biofilm matrix	Kreth et al., 2009; Jack et al., 2015
*Streptococcus intermedius*	Unknown	Unknown	Biofilm matrix	Petersen et al., 2004; Nur et al., 2013
*Streptococcus mutans*	Vesicles	Unknown	Biofilm matrix	Liao et al., 2014
*Streptococcus pneumoniae*	Competence-induced lysis	QS (CSP) (competence)	Biofilm matrix, HGT, nutrient source?	Steinmoen et al., 2002; Moscoso et al., 2006
*Streptococcus sanguinis*	Lytic-independent mechanism	Unknown	Biofilm matrix (aggregation)	Kreth et al., 2009
**ARCHAEA**				
*Halorubrum lacusprofundi*	Unknown	Unknown	Biofilm matrix	Fröls et al., 2012
*Haloferax volcanii*	Unknown	Unknown	Biofilm matrix, HGT, nutrient source	Chimileski et al., 2014a,b
*Thermococcus* spp.	Vesicles/unknown	Unknown	Unknown	Soler et al., 2008
*Thermococcus onnurineus*	Vesicles	Unknown	Unknown	Choi et al., 2015
**EUKARYOTES**				
*Aspergillus fumigatus*	Autolysis	Unknown	Biofilm matrix (antifungal resistance)	Rajendran et al., 2013
*Candida albicans*	Unknown	Unknown	Biofilm matrix (antifungal resistance)	Kasai et al., 2006; Martins et al., 2010; Mathé and Van Dijck, 2013

*We have listed all the microorganisms that have been described to date that release eDNA. However, it is important to note that only a small proportion of the microbial species present on Earth can be grown under laboratory conditions. Therefore, if we take into account the widespread distribution of eDNA in the environment it is very likely that the list is small compared to the real number of living microorganisms able to produce eDNA*.

### Neisseria gonorrhoeae

The genus *Neisseria* includes species that have co-evolved with humans. While most of the species in this group inhabit the human nasal pharynx, *N. gonorrhoeae* is a gram-negative human obligated pathogen that infects the genital tract (Dillard and Seifert, 2001). This bacterium can release large amounts of DNA for natural transformation into the medium, during exponential phase, unrelated of cell lysis (Dillard and Seifert, 2001), by using an active eDNA release mechanism (Hamilton et al., 2005). *N. gonorrhoeae* carries a genetic island named gonococcal genetic island (GGI) encoding a type IV secretion system (T4SS), by which chromosomal DNA is secreted to the extracellular medium. The GGI is 57 kbp long and is integrated in the replication terminus of the chromosome (Dillard and Seifert, 2001; Hamilton et al., 2005). The T4SS has been found both in gram-positive and gram-negative bacteria. They are conjugative or effector systems that translocate DNA-protein complexes or proteins (Alvarez-Martinez and Christie, 2009). It is notheworthy that T4SS from *N. gonorrhoeae* is the first described to be involved in DNA release into the extracellular medium in a cell contact independent manner (Hamilton et al., 2005). Moreover, it has been demonstrated that this process is completely independent of autolysis (Dillard and Seifert, 2001).

The DNA secreted by the T4SS is single stranded (ssDNA) with its 5′ end blocked, probably by a relaxase encoded by *traI*, a gene present in the GGI, which together with *traD* (a putative coupling protein) are suspected to regulate the T4SS activity (Salgado-Pabón et al., 2007; Ramsey et al., 2011). *N. gonorrhoeae* has natural competence with high transformation rates (Dillard and Seifert, 2001; Hamilton and Dillard, 2006), and takes up only DNA with a specific 10 bp sequence, typical from *Neisseria* (Goodman and Scocca, 1988; Hamilton and Dillard, 2006). Its high DNA interchange capacity is necessary to generate antigenic diversity in order to make the infection persist in the human population. In line with this, Hamilton and Dillard (2006) demonstrated that the DNA secreted by the T4SS is effective for natural transformation in gonococcus, thus contributing to HGT (Ramsey et al., 2011). Moreover, Dillard and Seifert (2001) reported that the transformation efficiency was higher with DNA secreted by the T4SS than with DNA released by autolysis. *N. gonorrhoeae* may use this secretion system instead of cell lysis to avoid the host immune system activation. Even if DNA is a pro-inflammatory agent, its effect can be blocked by methylation and in fact the eDNA secreted by the T4SS is highly methylated chromosomal DNA, thus supporting this theory (Hamilton et al., 2005).

### Pseudomonas aeruginosa

**Figure 1:**
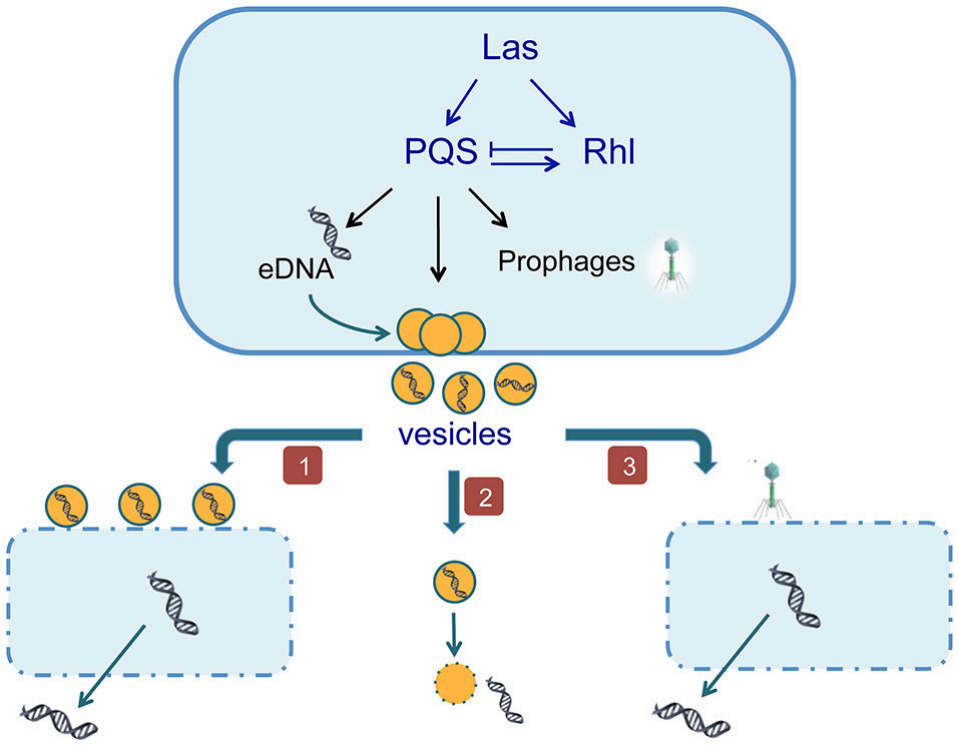
The eDNA production in *Pseudomonas aeruginosa* is induced by QS signals. (1) Vesicles cause lysis of other cells of the same culture; (2) the secreted vesicles carry the eDNA across the membrane to the extracellular medium; (3) QS activates the induction of prophages causing the death of neighboring cells.

*P. aeruginosa* is an opportunistic gram-negative pathogen that can proliferate in several hosts such as plants, insects, nematodes, and mammals (D'Argenio et al., 2002). This bacterium is able to release a large amount of double stranded genomic DNA (up to 18 μg ml^−1^) under QS control, in late-logarithmic growth phase when a certain cell density is reached in the liquid culture. In addition, there is a basal level of eDNA which production is independent of QS signals (Allesen-Holm et al., 2006). QS is a cell density-dependent communication system, which regulates cooperative behaviors. In *P. aeruginosa* there are tree QS systems, namely: *las, rhl*, and *pqs* (Pseudomonas quinolone signal; Wilder et al., 2011). All of them, and in particular the *pqsA* gene, which stimulates the eDNA production, and *pqsL*, which inhibits it, are involved in eDNA release (Allesen-Holm et al., 2006). Basically, the *las* system regulates the two others (Latifi et al., 1996; Pesci et al., 1997), and while *rhl* inhibits *pqs, pqs* is able to activate *rhlI*. At the same time *pqs* regulates eDNA release, membrane vesicles production and prophage induction (Figure 1; Froshauer et al., 1996; Mashburn and Whiteley, 2005; Allesen-Holm et al., 2006).

Cell lysis was proposed as the mechanism of eDNA release in *P. aeruginosa* based on the presence of an intracellular enzyme, β-galactosidase in the extracellular medium (Allesen-Holm et al., 2006). Then, a subpopulation is lysing, and it was sugested that vesicles released by some cells are causing the lysis of their siblings. This hypothesis is based on the fact that *P. aeruginosa* releases during exponential growth phase membrane vesicles (MVs) filled with lipopolysaccharides, hydrolytic enzymes, virulence factors and DNA (Kadurugamuwa and Beveridge, 1996). These vesicles are able to fuse with the outer membrane of gram-negative bacteria and to adhere to gram-positive bacteria (Kadurugamuwa and Beveridge, 1996), and they have been considered as part of a predation process (Nakamura et al., 2008). Currently it is considered that MVs are responsible for eDNA presence in the medium whether by its own lysis with the consequent eDNA release or by causing the lysis of a subpopulation of cells (Allesen-Holm et al., 2006). As in the case of eDNA release, it has been demonstrated that MVs production is not a random process but is dependent on QS signals. Specifically, it depends on the *las* and *pqs* systems (Renelli et al., 2004; Nakamura et al., 2008). The *pqs* system is not only implicated in eDNA production, as quinolones are well-known for triggering prophage induction and cause cell lysis (Froshauer et al., 1996). Considering that the motility is necessary for phages propagation, Allesen-Holm et al. (2006), analyzed a motility mutant and concluded that the PQS mediates prophage induction and the flagella/pili-dependent phage propagation are involved in eDNA release.

Interestingly, it has been recently discovered that *P. aeruginosa* can release eDNA through explosive cell lysis under stress conditions, and that these explosive events also produce MVs through vesicularization of shattered membranes. However, it is still not clear if under normal conditions this process is due to fratricide or to altruistic suicide (Turnbull et al., 2016).

### Streptococcus pneumoniae

eDNA release has also been described in gram-positive bacteria, for instance in the case of the human pathogen *Streptococcus pneumoniae*. This microorganism asymptomatically colonizes the upper respiratory tract to lately be potentially the cause of different diseases such as pneumonia, meningitis and sepsis in old and young people as well as in immunosuppressed patients. Besides, it is the main cause of middle ear infections in children (Moscoso et al., 2006).

The induction of the competent state in this bacterium triggers the genomic DNA release in a subpopulation of cells by lysis. In liquid cultures, 5–20% of the cells in a competent population will lyse and act as donors of DNA (Steinmoen et al., 2002). In all *Streptococcus* sp. of the mitis phylogenetic group, competence is induced by the pheromone CSP (competence-stimulating peptide), which has to reach a critical extracellular concentration (ranging from 1 to 10 ng ml^−1^) and be accompanied of a population density of 10^7^ cells ml^−1^ in order to activate the process. The primary structure of the CSP molecule varies depending on the species, ensuring that the communication is mainly intraspecific. In *S. pneumoniae* eDNA production is therefore a process dependent on QS signals, where a certain cell density is needed in order to develop that social behavior, maybe because cell-to-cell contact is necessary or because during the stationary phase some peptidoglycan variations in the cell wall may facilitate the cell lysis, as explained in detail below (Tomasz, 1966; Håvarstein et al., 1995; Steinmoen et al., 2002, 2003; Moscoso and Claverys, 2004).

**Figure 2:**
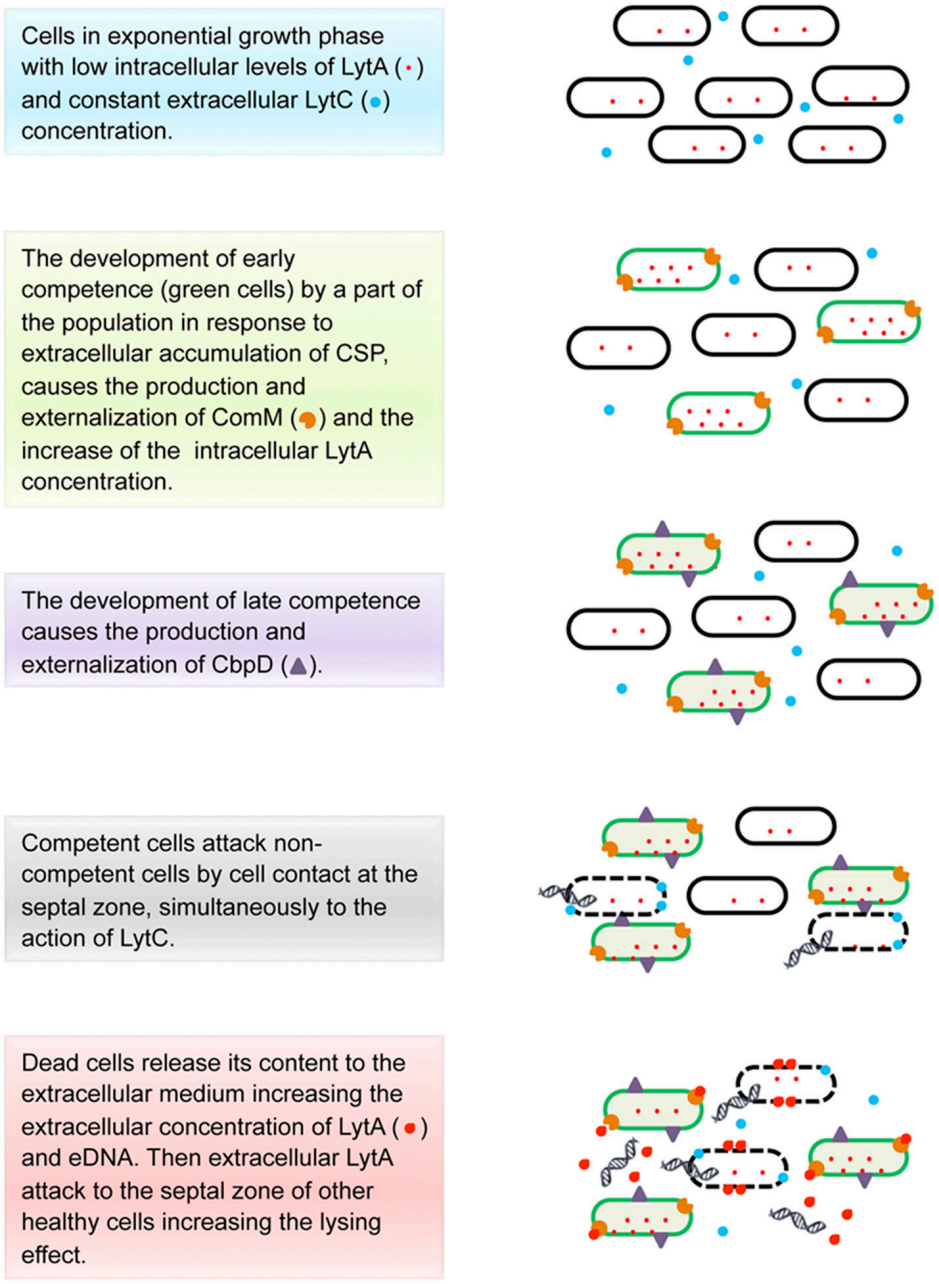
Model for eDNA production in *Streptococcus pneumoniae*.

*S. pneumoniae* releases to the extracellular medium 0.5% of the total chromosomal DNA of the culture (Moscoso and Claverys, 2004) by a fratricide or predatory lysis mechanism activated by CSP (Eldholm et al., 2009) and mediated by the binding-choline D proteins: CbpD, LytA, and LytC (Steinmoen et al., 2002; Kausmally et al., 2005; Eldholm et al., 2010; Berg et al., 2012; Mellroth et al., 2012; Figure 2). The competence state is triggered by the pheromone CSP that is detected by ComD, a membrane histidine kinase receptor, which in turns phosphorilates ComE, thereby activating the early competence genes, in particular *comX*, responsible for the activation of the late competence genes. Interestingly, the genes encoding CbpD and LytA form part of the *comX* regulon (Håvarstein et al., 1995; Steinmoen et al., 2002; Berg et al., 2012). CbpD is a murein hydrolase no covalently anchored to the secreting cells wall (Kausmally et al., 2005; Eldholm et al., 2010) and considered the main protein needed for the fratricide behavior (Kausmally et al., 2005). CbpD is only produced by competent cells (Eldholm et al., 2009), being able to cause the lytic effect by itself, even if its action is enhanced in the presence of LytA and LytC (Eldholm et al., 2009; Wei and Håvarstein, 2012). The lysis takes place by cell-to-cell contact through the disruption of the sibling cells at the septal zone. This process is only effective when the competent cells interact with cells from the same or closely related species that carry choline-decorated teichoic acids in their cell walls (Eldholm et al., 2010).

Taken together, data had led to a model of fratricide action that is still incomplete but it provides an approximate idea of what is happening (Figure 2). To understand this model it is noteworthy to mention that the composition and chemical structure of the peptidoglycan within the cell walls plays a critical role since the activity of both CbpD and LytA [a virulence factor involved in both the fratricidal- and penicillin-lysis processes, (Steinmoen et al., 2002)], would depend on it (Eldholm et al., 2010; Mellroth et al., 2012). According to Mellroth et al. (2012), during the exponential growth, where the cells are actively dividing, most of the LytA protein is intracellular and the small portion anchored to the extracellular side of the cell wall might not be able to cause its lytic effect because of the presence of the active cell wall synthesis machinery. Then, at the onset of stationary phase, several changes happen. First, the high population density triggers the release of CSP, which in turn activates the early competence in a subpopulation of cells (thereby competent cells produce ComM—an integral membrane protein that gives protection against the action of choline-binding proteins (Kausmally et al., 2005; Wei and Håvarstein, 2012)—becoming immune to the later lytic event, and then activates the late competence (Håvarstein et al., 1995; Steinmoen et al., 2002; Eldholm et al., 2009; Berg et al., 2012). This last step causes the overproduction of LytA (Steinmoen et al., 2002) and the synthesis and externalization of CbpD by competent cells (Eldholm et al., 2009). It has been proposed that CbpD together with LytC (a cell-wall lytic enzyme present in the extracellular medium, which synthesis is not regulated by competence; Eldholm et al., 2009; Wei and Håvarstein, 2012) cause the lysis of the non-immunized cells, attacking the septal zone of the non-competent cells and enabling the release of the cytoplasmic LytA to the extracellular medium (Eldholm et al., 2009; Mellroth et al., 2012). Considering that at that point the cell growth is arrested and the cell wall machinery has become inactive, LytA is supposed to have access to its target, the nascent peptidoglycan region (in the septal zone), degrading the neighboring cell walls, thereby generating a lytic cascade leading to the accumulation of extracellular LytA and reaching the threshold necessary to activate an autolytic effect (Kausmally et al., 2005; Eldholm et al., 2009; Mellroth et al., 2012). Finally, the content of the disrupted cells might cause a virulent effect on the host as a result of the activation of the immune response, while eDNA is uptaken by competent cells, in a process called transformation, promoting HGT (Claverys et al., 2008; Johnsborg and Håvarstein, 2009; Muschiol et al., 2015; Wholey et al., 2016).

### Bacillus subtilis

**Figure 3:**
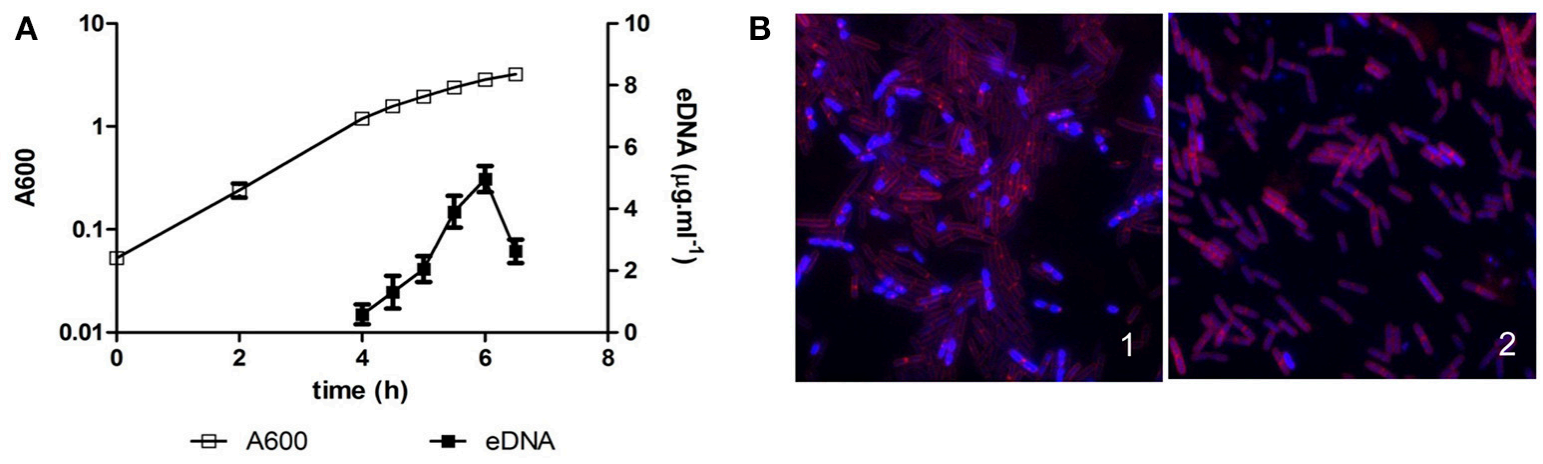
eDNA production in *Bacillus subtilis* 3610. **(A)** Batch culture of strain 3610 in MSgg at 37°C with aeration. A600 refers to the absorbance of the culture at 600 nm, and eDNA refers to the concentration of eDNA in the culture supernatant. eDNA is released during the transition from exponential to stationary phase, followed by a rapid decrease in eDNA concentration. The data presented are representative of the results obtained in ten different experiments. **(B)** fluorescent microscopy image of cells from a wild type 3610 strain (1) and an eDNA defective mutant (2) after 5 h 30 min in batch growth. The DNA is stained blue (DAPI) and cellular membranes are stained red (FM4.64). A subpopulation of cells has a greater intensity of DNA staining in a wild type strain but not in a defective mutant in eDNA production.

*B. subtilis* is a gram-positive, non-pathogenic, bacterium isolated from a wide range of environments, both aquatic and terrestrial, sometimes associated with roots or in animal dregs (Earl et al., 2008). eDNA was first reported to be released by different laboratory strains, such as the 168 strain, during exponential and early stationary phase (Sinha and Iyer, 1971; Lorenz et al., 1991), and this eDNA was proposed to have a role in HGT (Crabb et al., 1977; Lorenz et al., 1991). However, *B. subtilis* laboratory strains have lost some social behaviors as a result of genetic modifications and pressure selection that facilitated their manipulation in the laboratory. For instance, the undomesticated strain 3610, which is the ancestor of most of the laboratory strains (Burkholder and Giles, 1947; Kunst et al., 1997), is able to develop social behaviors such as the formation of multicellular aerial structures (Branda et al., 2001) or social motility named swarming (Kearns and Losick, 2003). Thus, the production of eDNA was recently explored in the 3610 strain, and it was shown that a large amount of eDNA (as compared to previous studies based on laboratory strains) is released during the transition from exponential to stationary phase, then followed by a fast decrease in eDNA concentration (Figure 3A; Zafra et al., 2012). This eDNA was found to be fragmented in a size range from 10 Kbp to 400 bp, it matches the complete genome as shown by using oligonucleotide microarrays, and it could be synthesized by a normal replicative machinery, not an error-prone polymerase (Zafra et al., 2012). Similarly as proposed for the laboratory strains (Sinha and Iyer, 1971; Lorenz et al., 1991), the 3610 strain produces eDNA in a lysis-independent way (Zafra et al., 2012). Thus, eDNA must be released by an active mechanism, maybe such as the type IV secretion system described for other gram-positive bacteria as *Neisseria* (Hamilton et al., 2005), or through association with vesicles. The latter hypothesis is based on the recent discovery of vesicles production both by 168 and 3610 strains (Brown et al., 2014). The content of these vesicles has not yet been described, thus it may be possible that they carry eDNA as *P. aeruginosa* vesicles (Kadurugamuwa and Beveridge, 1996).

As reported in *P. aeruginosa* and *S. pneumoniae*, eDNA production in *B. subtilis* depends on QS signals involved in the early stages of the development of competence. The 3610 strain allowed a visual screening to search for mutants affected in eDNA production. On solid rich medium, the colonies formed by this strain are small and do not spread properly over the surface. However, spontaneous mutants emerge from these colonies, exhibiting an extended morphology, and interestingly, these mutants were defective in eDNA release (Zafra et al., 2012). Thus, a screening was based on the search for extended colonies in a transposon mutant library of the strain 3610, assuming that the spread morphology could be linked to the defect in eDNA production. Many of the transposons were inserted in the *oppA, oppF*, and *comXP* genes, which are QS systems involved in the early stage of competence (Zafra et al., 2012). *oppA* and *oppF* encode components of the oligopeptide permease Opp, an ABC transporter involved in the active import of signal oligopeptides into the cell (Lazazzera, 2001; Solomon et al., 2003), such as CSF (competence and sporulation factor). On the other hand, ComX is another QS signal that activates ComP. Both signals, CSF and ComX induce the activation (phosphorylation) of the response regulator ComA (Solomon et al., 1995; Solomon and Grossman, 1996). Phosphorylated ComA activates the operon *srf-comS*, which encodes the surfactin synthetase (SrfAA, SrfAB, SrfAC, and SrfAD) and ComS, the first signal of the late stage of competence. The small gene *comS* is located within the *srfAB* gene, in a different open reading frame (D'Souza et al., 1994). ComS controls ComK levels, which is the regulator of the late competence genes. It was also shown that a *comA* mutant is also defective in eDNA release (Zafra et al., 2012). However, mutants affected in late competence, such as *comK* and other genes related to DNA uptake machinery (*comEA* and *comGA*) are not affected in eDNA production (Zafra et al., 2012). Thus, there is a divergence in the development of natural competence and eDNA release at this stage. Indirect evidence supports a possible role of ComS in eDNA release. A mutation in the first gene of the operon encoding the surfactin synthetase, *srfAA* affects the release of eDNA, but surfactin production was not affected in other mutants defective in eDNA production. Hence, the transcription of *comS*, which is inside the *srfAB* gene in a different open reading frame, could be affected by a polar effect of the *srfAA* mutation (Zafra et al., 2012). Thus, ComS could be involved in the divergence of both competence and eDNA production pathways.

Interestingly, eDNA appears to be released by a subpopulation of cells in *B. subtilis*. As aforementioned, cell lysis is not involved in the production of eDNA in 3610 strain, therefore, a higher level of DNA replication in the producer cells might occur. In fact, it was shown by fluorescence microscopy and flow cytometry that a subpopulation of cells of about 10–15% has increased DNA staining intensity in a wild type strain but those cells were not observed in defective mutants in eDNA production (Figure 3B; Zafra et al., 2012).

## Biological functions of eDNA

eDNA can be used by bacteria for several key functions, such as structural component of biofilms, nutrient source, and in HGT. Our aim is to show its importance in the natural life cycle of bacteria emphasizing the social and multicellular lifestyle. It is noteworthy that regulatory networks associated with different eDNA functions are linked to QS and social behaviors in some bacteria (Spoering and Gilmore, 2006; Vorkapic et al., 2016).

### eDNA function in biofilms

Most microorganisms in natural environments do not operate as individual cells, but are organized in multicellular communities called biofilms (Costerton et al., 1999; Hall-Stoodley et al., 2004; Tang et al., 2013). The cells in these communities are protected against chemical and physical stresses, challenging environmental conditions or even predators (Mah and O'Toole, 2001; Matz and Kjelleberg, 2005; Anderson and O'Toole, 2008), and the exchange of genetic information is favored. The architecture of structured multicellular communities depends on the production of an extracellular matrix, which is usually formed by exopolysaccharides, proteins, and DNA (Sutherland, 2001; Flemming and Wingender, 2010). Interestingly, many microorganisms release eDNA within their biofilms. For instance, *N. gonorrhoeae* (Steichen et al., 2011), *P. aeruginosa* (Whitchurch et al., 2002) and *P. chlororaphis* (Wang et al., 2016), some *Staphylococcus* species such as *S. epidermis* (Qin et al., 2007) or *S. aureus* (Rice et al., 2007), *S. pneumoniae* (Moscoso and Claverys, 2004), *Enterococcus faecalis* (Thomas et al., 2008), *Helicobacter pylori* (Grande et al., 2015), and *Campylobacter jejuni* (Svensson et al., 2014). Thus, the presence of eDNA in biofilms is definitely a widespread feature (Okshevsky and Meyer, 2015; Vorkapic et al., 2016). The proposed functions for eDNA in the biofilms are: (i) structural component within the biofilm that provides stability to the entire structure (Moscoso and Claverys, 2004; Allesen-Holm et al., 2006; Thomas et al., 2008; Lappann et al., 2010; Wang et al., 2016); (ii) a factor that promotes the formation of biofilm and the production of extracellular matrix (Qin et al., 2007; Barken et al., 2008; Zweig et al., 2014); and (iii) a role in gene transfer through transformation of competent sister bacteria (Springael et al., 2002; Molin and Tolker-Nielsen, 2003).

The first description that eDNA is essential for biofilm formation was in *P. aeruginosa* (Whitchurch et al., 2002) and other studies demonstrated its structural role in different microorganisms (Whitchurch et al., 2002; Hall-Stoodley et al., 2004; Qin et al., 2007; Liu et al., 2008; Seper et al., 2011). Even if eDNA is the most abundant polymer within the *P. aeruginosa* matrix (Matsukawa and Greenberg, 2004), it is still unclear whether or not it is essential for the proper biofilm development (Whitchurch et al., 2002; Nemoto et al., 2003; Matsukawa and Greenberg, 2004). While DNAase I treatment seems to affect early stages of biofilm formation, mature biofilms are immune, thus implying different functions of eDNA during the development of the biofilm (Whitchurch et al., 2002). Nevertheless, other research groups have found that eDNA is not necessary for normal biofilm formation (Nemoto et al., 2003; Matsukawa and Greenberg, 2004), concluding that discrepancies may be due to the use of different strains. Despite the unclear relevance of the eDNA for *P. aeruginosa* biofilm formation, it is considered as an adhesion compound that enables cell-to-cell attachment, even in planktonic cultures, stabilizing the biofilm and providing resistance against degrading agents such as the SDS detergent (Klausen et al., 2003; Allesen-Holm et al., 2006). Moreover, it has been proved that eDNA can establish ionic interactions with Pel, a protein that plays a role in maintaining cell-to-cell interactions within biofilms and contributes to antibiotic resistance; however, its relevance may vary by strain (Jennings et al., 2015).

**Figure 4:**
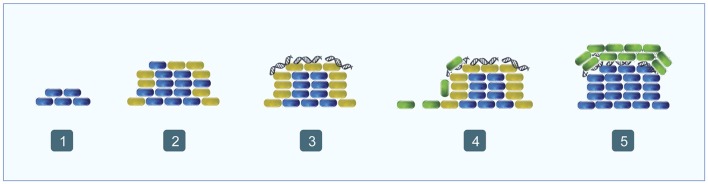
Biofilm formation in *Pseudomonas aeruginosa*. (1) Non-motile cells begin to accumulate forming the stalk (blue); (2) most of the outer cells express the PQS system (orange); (3) eDNA begins to accumulate over the stalk forming ring-like shaped structures (orange fibers); (4) motile cells migrate toward the top the stalk (green); (5) motile cells form the cap (green).

Biofilm formation in *P. aeruginosa* is a sequential process characterized by a high population diversity (Figure 4). The first step in the process is the formation of microcolonies, starting with the development of the stalk, which is made by non-motile cells that aggregate. During these early steps, eDNA is widely spread along the surface of the substrate and facilitates efficient traffic flow throughout the furrow network by maintaining coherent cell alignments, thereby avoiding traffic jams and ensuring an efficient supply of cells to the migrating front (Klausen et al., 2003; Allesen-Holm et al., 2006; Gloag et al., 2013). Then begins the formation of the upper part of the mushroom-like structure, called the cap. This process is characterized by the migration of a motile subpopulation in a QS dependent way, and type IV pili, flagella and eDNA are involved. Basically, motile cells are believed to use the type IV pili structure to interact with the eDNA and to move toward the top of the stalk using swimming or swarming/twitching motility (Figure 4; Klausen et al., 2003; Allesen-Holm et al., 2006; Barken et al., 2008). This hypothesis is supported by the fact that the eDNA distribution changes during biofilm maturation and at that point is concentrated in the external part of the stalk, between the upper part of the stalk and the cap (Allesen-Holm et al., 2006). These results agree with the observation that only the cells located in the most external part of the stalk are expressing the PQS system (Yang et al., 2007). Moreover, the QS system is also regulating the synthesis of surfactants, necessary for flagellar motility and probably promoting the migration of cap-forming cells (Klausen et al., 2003; Barken et al., 2008). Finally, in mature biofilms, the eDNA is distributed in discrete, ring-shaped layers (Allesen-Holm et al., 2006). Interestingly, some strains develop an autolysis process in the center of mature colonies, which might be another source of eDNA (Berk, 1965; D'Argenio et al., 2002). Overall, these studies point to the idea that eDNA is a chemoattractant or a structural polymer that the motile cells need to migrate in a controlled way to the top of the stalk and to form the structure of the cap.

eDNA is also an abundant component of the extracellular matrix, together with proteins and exopolysaccharides, of the biofilms formed by *S. pneumoniae* even although its role remains unclear (Moscoso et al., 2006; Muñoz-Elías et al., 2008; Domenech et al., 2013). The mechanism of eDNA release within *S. pneumoniae* biofilms is unknown. It appears to be released spontaneously (Moscoso et al., 2006), maybe as a result of the action of bacteriophages (Carrolo et al., 2010), a speculation that agrees with previously reported suicidal behavior (McCarty, 1985). Interestingly, the lytic enzymes necessary for eDNA release in planktonic cultures (LytA and LytC) appear to be involved in the correct biofilm formation, however its role is not yet understood (Moscoso et al., 2006). Subsequently, it was shown by confocal-laser microscopy that the LytC autolysin can interact with eDNA. LytC-eDNA complexes may contribute to the production of an insoluble nucleoproteins network in the extracellular matrix, which in turn acts as a connector between cells and between cells and substrate (Domenech et al., 2013). In addition, other choline binding proteins such as LytA, LytB, Pce, PspC, or CbpF have been proved to interact with eDNA, independently of its enzymatic activity (Domenech et al., 2012, 2013). Moreover, both LytA and C mutants have problems forming normal biofilms (Moscoso et al., 2006), therefore, autolysins that were shown to be involved in eDNA release in planktonic cultures could also be involved in eDNA release in biofilms.

In *N. gonorroheae*, the presence of eDNA within the biofilm is independent of the T4SS, the mechanism described for eDNA release in planktonic cultures, and strains lacking this system can form normal biofilms (Greiner et al., 2005). On the other hand, ssDNA is necessary for the early stages of gonococcal biofilm formation in a different strain (MS11; Zweig et al., 2014). Interestingly, a type of vesicles called blebs are formed on the membrane surface of the gonococcus bacteria as they grow in biofilms, independently of cell lysis, and they are filled with plasmid or chromosomal DNA (Dorward et al., 1989; Greiner et al., 2005). All gonococcal strains produce blebs believed to form part of the membranous structures visualized in the biofilm using microscopy techniques (Greiner et al., 2005). These structures are considered to provide physical support to the biofilm structure and are thought to be the source of eDNA present in biofilms.

*B. subtilis* is a gram-positive model bacterium for biofilm formation and the undomesticated strain 3610 is able to construct multicellular aerial structures or fruiting bodies in which spores form (Branda et al., 2001; Hamon and Lazazzera, 2001). However, eDNA actively released at the end of exponential phase is not required for biofilm and fruiting body formation in 3610 strain, since mutants defective in eDNA production are able to form the same robust biofilms and aerial structures as the wild type strain (Zafra et al., 2012). On the other hand, it has been suggested that DNA released by lysis mediated by cannibalism during stationary phase (González-Pastor et al., 2003) could have a strong relationship with matrix development during biofilm establishment (López et al., 2009). In any case, the presence of eDNA in the extracellular matrix of *B. subtilis* biofilms has not yet been shown.

eDNA has a role in the initial steps of biofilm formation in other microorganisms. In *Staphylococcus epidermidis* eDNA increases adhesion and aggregation through acid-base interactions (Das et al., 2010), and in *Streptococcus mutans*, could mediate adhesion to a hydrophobic surface (Das et al., 2011). In *Listeria monocytogenes*, the binding of eDNA to peptidoglycan is involved in adhesion, but N-acetylglucosamine is also required (Harmsen et al., 2010), and in *Bacillus cereus*, eDNA produced by a planktonic culture associates with the cell surface and mediates adhesion with the substrate, which initiates biofilm formation and acts as protective shield against some antimicrobials. In this case adenylosuccinate synthetase activity (purine biosynthesis) is required for biofilm formation, but normal growth is not impaired in *purA* mutants (Vilain et al., 2009).

Extracellular enzymes as nucleases also play a role in the regulation of eDNA and hence in the formation or structure of the biofilm. The extracellular nucleases Dns and Xds in *Vibrio cholerae* are able to influence the structure, detachment and dispersion of biofilms by modulation of eDNA (Seper et al., 2011), and a thermonuclease in *S. aureus* is involved in the control of lysis and eDNA release during biofilm development and in promoting biofilm dispersal (Mann et al., 2009). Interestingly, *S. aureus* biofilms are structured with a skeletal framework composed of eDNA covalently interacting with the beta-toxin, a neutral sphingomyelinase and a virulence factor (Huseby et al., 2010). *E. faecalis* is also able to regulate the eDNA release by autolysis to influence biofilm development (Thomas et al., 2008, 2009). In addition, two extracellular endonucleases, ExeS and ExeM are involved in eDNA degradation in biofilms formed by *Shewanella oneidensis* MR-1, which might enable cell detachment (Gödeke et al., 2011a).

eDNA also has a role in biofilm dispersal. For instance, in *Caulobacter crescentus* the eDNA could inhibit the deposition of swarmer cells in a way that they are not able to settle down in an existing biofilm, but in a new location (Berne et al., 2010). In *N. meningitidis* biofilms a colonizing population is able to form biofilms containing eDNA and a high transmission population that is poorly associated due to the absence of eDNA (Lappann et al., 2010).

In relation to biofilms, eDNA might also be involved in defense mechanisms and therefore in the virulence of pathogenic microorganisms. eDNA plays its protective role by reducing the transport of antimicrobials through the matrix or by interactions with other matrix compounds (Flemming and Wingender, 2010). But also, eDNA is able to chelate cations, for instance in *P. aeruginosa* when magnesium is chelated, a genetic program that modify its surface and that could increase pathogenicity or antimicrobial resistance is triggered. Furthermore, the acidification produced by eDNA is a signal to induce resistance to antimicrobial peptides (Mulcahy et al., 2008; Lewenza, 2013; Wilton et al., 2015).

In addition, other studies have reported the presence of eDNA in biofilms formed by other microorganisms. In the archaea *Haloferax volcanii* eDNA was detected in biofilms and it was suggested that it is related to the formation of biofilms, social motility, HGT and phosphorous source (Chimileski et al., 2014a,b). Also some fungal microorganisms as *Candida albicans* and *Aspergillus fumigatus* might use eDNA to form biofilm and to enhance antifungal resistance (Martins et al., 2010; Mathé and Van Dijck, 2013; Rajendran et al., 2013).

### eDNA function in DNA damage repair and horizontal gene transfer

The uptake of environmental DNA by some bacteria was thought to have evolved as a primitive sex to repair damaged genomes and to avoid Muller's ratchet and keep up in evolutionary arms race in small populations. In *B. subtilis* the SOS pathway (a global response to DNA damage) were induced both by DNA damaging agents and by competence development (Love and Yasbin, 1984; Love et al., 1985; Lovett et al., 1989; Prudhomme et al., 2006). DNA uptake was not regulated by DNA damage (Redfield, 1993; Redfield et al., 1997), but in *B. subtilis*, the SOS system is coordinated with eDNA uptake to identify homologous regions in the genome and recombine them in order to incorporate any benefit it provides (Kidane et al., 2009, 2012; Cárdenas et al., 2012). The master regulator of competence ComK initiates the K-state (competence) differentiation by controlling the expression of a wide variety of clusters some of which relate to eDNA uptake and others to repair and recombination (*recA, dinB*; Berka et al., 2002; Hamoen et al., 2002; Ogura et al., 2002). During K-state, the recombination machinery is dynamically associated with DNA uptake system (Tadesse and Graumann, 2007; Hahn et al., 2009) providing different proteins that are required, depending on the type of DNA acquired (chromosomal, plasmid or viral; Kidane et al., 2012; Carrasco et al., 2016). In contrast, other bacteria lack a “*bonna fide*” SOS response, and eDNA uptake could be useful for repairing mutations in the genome, in these cases there is an induction of competence after DNA damage (Redfield, 2001; Claverys et al., 2006, 2008; Engelmoer and Rozen, 2011). For example, in *S. pneumoniae*, an SOS-like system has not been found, and in this case competence replaces this repair pathway. Mitomycin C treatment causes DNA damage and induces the competence regulatory cascade, which includes expression of the *recA* gene, making DNA repair possible or acquiring suppressor mutations that maintain the fitness of the population (Prudhomme et al., 2006). As *S. pneumoniae* is a well-known human pathogen, the practical implications of this work are interesting: treatment with bacteriolytic antibiotics should be implemented with competence inhibitors to prevent eDNA uptake, which could favor the survival of the pathogen. *N. gonorrhoeae* is another example of a human pathogen that takes advantage of a common extracellular genetic pool, not only to share antibiotic resistance, but also to enhance pilin antigenic variation through allele recombination with acquired eDNA from the environment (Seifert et al., 1988; Gibbs et al., 1989; Davies, 1994).

During harsh conditions, microorganisms are able to increase their survival through different strategies such as error-prone repair, fluctuations of some regulators (Moxon et al., 1994) and increased eDNA uptake to improve genomic adaptability of the community (Feil et al., 1999; Donati et al., 2010; Mell et al., 2011). Szöllősi et al. (2006), described a computer model based on natural populations with weak migration and environmental fluctuations. In this case, the eDNA pool acted as a collective reservoir of lost functions that could be quickly recovered by DNA uptake when the physical conditions demand its reloading. Undoubtedly, this study promotes the view of eDNA as a social deposit of genetic information even when it was only an *in silico*-based hypothesis. Levin and Cornejo (2009), completed the computer simulations with genetic transfer rates of known bacteria as *B. subtilis, H. influenza*, and *S. pneumoniae* showing that under realistic conditions the exchange of genetic information within populations is an evolutionary advantage and provides higher rates of evolution. In fact, computer modeling has been so finely tuned that supports the evidence that only a small subpopulation of *B. subtilis* becomes competent. If the genetic benefits of extracellular DNA uptake were so advantageous, the question that remains is why only a small fraction of the population [10% in the case of *B. subtilis* (Smith et al., 1981)] develops this process. Wylie et al. (2010), introduced in the equation the “persistence” phenotype of the competent subpopulation (reduced metabolic rates of competent cells compared with non-competent ones (Nester and Stocker, 1963; Haijema et al., 2001; Briley et al., 2011; Hahn et al., 2015), and it turned out that the potential benefits of eDNA uptake counterbalance the reduced replication rate, and this dilemma was solved in divided subpopulations. In the case of *B. subtilis* the development of this differentiation is based on a molecular fluctuation (bistability) of the master regulator ComK (Solomon and Grossman, 1996; Macfadyen, 2000; Maamar and Dubnau, 2005).

Thus, acquisition of genetic information from an environmental pool is an evolutionary advantage, but it has some costs related to growth rates, limiting this process to a fraction of the population. This “social” genetic stock could not only provide information reloading, but also a way of generating diversity. Since DNA uptake must be followed by a recombination driven by the SOS system, it includes the possibility of an error-prone process.

In the bacterium *Acinetobacter baylyi*, short and damaged DNA sequences (>20 pb containing abasic sites, cross-links or miscoding lesions) could been taken up and integrated into the genome. This process is RecA-independent and is related to replication, probably through its use as primers in the lagging strand and the involvement of mismatch repair (Overballe-Petersen et al., 2013; Overballe-Petersen and Willerslev, 2014). Thus, eDNA could be relevant for evolution, as it makes possible the variability, independently of its length and quality. In the case of short and fragmented DNA, the result would be to generate genetic polymorphisms rather than the integration of new functions. Thus, we could be missing this role of eDNA, assigning the presence of polymorphisms to spontaneous mutations. Another important conclusion of this study is that eDNA molecules remain available as a genetic pool for bacterial populations for a long time and under harsh conditions.

Sexual recombination is a specialization in which genetic transfer is optimized to obtain only homologous DNA sequences. Thus, another step is required from the view of a common eDNA pool, and that could be the reason of some microorganisms to maintain some barriers to avoid random genetic transfer. For instance, *H. influenzae* only takes eDNA with specific marker sequences (Macfadyen, 2000) and *S. pneumoniae* coordinates the fratricide of a subpopulation with the competent state of the rest of the population (Steinmoen et al., 2002, 2003; Johnsborg and Håvarstein, 2009). In the case of *B. subtilis*, the active secretion of DNA in the medium is coordinated with competence development (Zafra et al., 2012). Both processes coincide physiologically in time and it has been shown that the active release of eDNA in *B. subtilis* populations is related to the regulatory pathway of early competence, since a *comA* mutant is defective in eDNA production, whereas mutations in late competence genes, such as those encoding the DNA uptake machinery, have no effects. In addition, eDNA release mutants are defective in competence. Finally, it was demonstrated that the *B. subtilis* eDNA is functional in HGT, which gives a rationale for this common regulation in the context of the coordination of both processes (Zafra et al., 2012). In *P. stutzeri* has also been suggested that certain cells donate DNA to the others through a mechanism that is not just cell lysis (Stewart et al., 1983). Some authors argue that barriers to random genetic exchange are necessary to develop sexual reproduction and that this is a prerequisite for multicellularity to evolve through stable cooperation of subpopulations and by limiting external interference (Overballe-Petersen and Willerslev, 2014). Therefore, *B. subtilis* stands out as a model for eDNA studies, since competence, controlled genetic transfer and differentiation in subpopulations are described (López and Kolter, 2010). Recently, an “*in silico*” study has shown that DNA uptake from an environmental pool of related sequences from killed siblings is able to control the spreading of mobile genetic elements, which could otherwise harm their bacterial hosts (Croucher et al., 2016).

### eDNA function as a source of nutrients

eDNA is an abundant compound in natural environments and is a potential source of energy and nutrients such as C, N and P. Specifically, orthophosphate is usually a limiting nutrient in a wide variety of environments. In aquatic ecosystems has been described that a large variety of microorganisms are capable to use eDNA as nutrient source (mainly as P-source), recycling and mineralizing it, but it does not seem to be a universal capability since the eDNA is abundant and persistent in the environment (Dell'Anno and Danovaro, 2005).

Research on aquatic systems has described the use of eDNA as energy and C source by a variety of bacteria, which can be classified into two groups depending on the molecular weight of the eDNA that they are able to use. They can usually grow with low molecular weight (LMW) or high molecular weight (HMW) eDNA molecules, however, very few bacteria are to use both molecules. Thus, there could be two strategies for using eDNA as C and energy source, and in natural environments, where the eDNA is recycled, a sequence of bacterial populations might appear. Bacteria able to grow with LMW eDNA could use extracellular nucleases to degrade it and take up the monomers (nucleosides or nucleotides) for their use. On the other hand, bacteria able to grow with HMW eDNA could uptake intact DNA molecules through competence, in which case the whole DNA molecule could be used as nutrient source or for genetic purposes (Lennon, 2007).

In several bacterial models nutrient limitation induces DNA uptake through competence gene cluster. In *Azotobacter vinelanii* it was described that glucose or ammonium ions in the culture medium repress transcription of competence genes whereas a nitrogen limiting culture activates both nitrogen fixation and competence (Page and Sadoff, 1976). Metal-reducing bacteria of the *Shewanella* genus could use eDNA to obtain orthophosphate (a limiting nutrient in the iron-rich environments in which they live) by releasing extracellular nucleases and phosphatases induced by low phosphate levels (Pinchuk et al., 2008; Gödeke et al., 2011a; Heun et al., 2012). Other phosphate-limited environment are hypersaline habitats where eDNA is present at high levels, the archeon *H. volcanii* can use it as nutrient source, mainly to obtain phosphate, but using DNA molecules with a specific methylation pattern (Zerulla et al., 2014; Chimileski et al., 2014a). In *H. influenzae* only the 15% of the radiolabelled eDNA is integrated in the genome by homologous recombination, the rest is degraded and used to synthesize different compounds (Pifer and Smith, 1985). In this microorganism, nutritional stress regulates competence through cAMP and catabolite regulator protein (CRP) protein (Macfadyen et al., 2001; Redfield et al., 2005). In *E. coli* natural competence has not been reported, however, this bacterium has eight genes homologous to the competence genes, which are related to the ability to grow during the long-term stationary phase in the presence of eDNA, an important skill for fitness in nutrient depleted cultures (Finkel and Kolter, 2001; Palchevskiy and Finkel, 2006). In addition, *P. aeruginosa* is able to release extracellular DNases under phosphate limiting conditions for the use of eDNA as nutrient source. This is important in virulence and biofilm formation as the lung in cystic fibrosis patients is a nutrient restrictive habitat where eDNA can be found as in other mucosal surfaces (Matthews et al., 1963; Mulcahy et al., 2010). In *V. cholerae*, another human pathogen, competence is regulated by a complex mixture of stimuli, some of them related to nutrient stress, and this regulation also plays an important role in virulence and biofilm formation. The intestine is a phosphate-limiting environment in which the expression of several extracellular nucleases is induced in this bacterium (Meibom et al., 2005; Antonova et al., 2012; McDonough et al., 2014, 2016).

Bacterial metabolism is a dynamic process in which recombination would sometimes be favored, or in other conditions, degradation to obtain resources would be a priority. Therefore, it is not necessary to exclude one funtion or another for eDNA, since both could be possible in different or even in the same microorganisms. For instance, in *B. subtilis* and *S. pneumoniae*, during eDNA uptake for recombination only one strand enters the cell and the other could be used as nutrient source, in contrast to *Haemophilus* or *Neisseria* that take up dsDNA (Stewart and Carlson, 1986; Dubnau, 1999). It should be noted that *B. subtilis* develops competence and eDNA release in the transition from exponential to stationary phase, when the culture medium begins to be depleted (Lorenz et al., 1991; Zafra et al., 2012). Nutrient-limited conditions activate the expression of competence genes in this microorganism (Redfield, 1993), and in fact, the master regulator CodY, involved in nitrogen metabolism, is able to regulate competence (Fisher, 1999). The internalized DNA strands, depending on their homology to the genomic DNA sequences and their modifications, could be found as recombined material and also as monomeric substrates to use during replication (Piechowska et al., 1975; Soltyk et al., 1975). On the other hand, the DNA strands outside the cell could be degraded by extracellular nucleases to be used as a source of nutrients.

## Concluding remarks: eDNA release as a social behavior

Overall, the results summarized in this review show that eDNA production is a widespread feature among living organisms, whereas the release mechanisms and functions of eDNA vary considerably depending on the species. It is of great interest that in most of microorganisms studied, eDNA production is an accurately regulated process in which DNA synthesis and release depends on the QS signals present in the media in response to cell growth. This is the case, for instance, of all species in which eDNA production is related to the development of competence such as *B. subtilis, S. pneumoniae, Staphylococcus lugdunensis, Streptococcus gordonii*, or *Acinetobacter calcoaceticus* (Palmen and Hellingwerf, 1995; Steinmoen et al., 2002; Zafra et al., 2012; Jack et al., 2015; Rajendran et al., 2015). In *B. subtilis* and *S. pneumoniae*, the pattern of competence development followed by the release of eDNA is almost the same. Considering that both species can be transformed with their own eDNA and that they share the signaling pathway for competence and eDNA production, this correlation has a significant relevance for HGT (Steinmoen et al., 2002; Zafra et al., 2012). In fact, it has been proposed that eDNA in these species is released correlating in time with the presence of cells of the same culture that are competent in order to ensure the intraspecific genetic flow, guaranteeing the adaptation and survival of the species. Interestingly, in *B. subtilis* and *S. pneumoniae* as well as in the case of *S. epidermis* or *E. faecalis*, only a fraction of the population produces eDNA, which implies the emergence of cell division of labor within the community (Steinmoen et al., 2002; Qin et al., 2007; Thomas et al., 2008; Zafra et al., 2012).

The differentiation of cells into tasks-specific groups is an essential feature of multicellular microbial communities (López and Kolter, 2010; González-Pastor, 2012; Zhang et al., 2012; van Gestel et al., 2015). The release of different signals into the extracellular medium directly affects the behavior of groups of cells in the population through changes in the expression of some of their genes, causing their differentiation and thereby resulting in the construction of multicellular communities (Camilli and Bassler, 2006). For instance, *B. subtilis* biofilms are composed of motile cells, sessile matrix producers and sporulating cells, specifically distributed within the multicellular structures (Branda et al., 2001; López and Kolter, 2010; González-Pastor, 2012). This cell differentiation has also been described in *P. aeruginosa* biofilms, where the eDNA plays an essential role and whose production is QS dependent (Allesen-Holm et al., 2006). Particularly in *N. gonorrhoeae, P. aeruginosa, C. crescentus*, and *C. jejuni* a relationship between eDNA production and motility has been highlighted, which again demonstrates the complexity of this process (Allesen-Holm et al., 2006; Berne et al., 2010; Salgado-Pabón et al., 2010; Svensson et al., 2014). In general terms, it is precisely during biofilm development that QS signals would play a critical role and their concentration gradients would affect such cellular responses. This might account for the observed differences in eDNA production between cells in planktonic cultures and in biofilms. In this sense, although several mechanisms were described for the eDNA release in planktonic cultures, some of them were not found or were different in biofilms (Sauer et al., 2002; Greiner et al., 2005; Allesen-Holm et al., 2006; Kim and Lee, 2016).

It is noteworthy that such discrepancies are not only due to different growth conditions and experimental procedures, but depends on the strains used in these studies. This is the case of *N. gonorrhoeae* in which most of the strains have different types of GGIs, while others lack them (Dillard and Seifert, 2001). The same diversity can be found in *P. aeruginosa* biofilms where the relevance of eDNA is unclear due mainly to differences between the strains (Nemoto et al., 2003; Matsukawa and Greenberg, 2004). It should be noted that in *B. subtilis* the differences between strains are determinant in their ability to develop certain social behaviors dependent upon QS, as demonstrated when comparing the undomesticated and the laboratory strains (Zafra et al., 2012). On the other hand, apart from QS signals, iron has been demonstrated to be essential in the modulation of eDNA production, for instance by triggering prophage induction in *S. oneidensis* (Binnenkade et al., 2014), mediating cell lysis during biofilm formation in *S. pneumoniae* (Trappetti et al., 2011) or regulating eDNA production by affecting the QS signaling system in *P. aeruginosa* (Yang et al., 2007).

Multiple functions have been described for eDNA, which makes it an even more interesting molecule that had already been considered. Not only can it be used as a nutrient but it has the ability to interact with other proteins as demonstrated for *P. aeruginosa, S. pneumonia*, or *S. intermedius*, functioning as a structural molecule for biofilm stability (Domenech et al., 2013; Jennings et al., 2015). Moreover, eDNA can be considered as a signaling molecule, suggested to form a macromolecular language or possibly to act as a chemoattractant (Allesen-Holm et al., 2006; Barken et al., 2008; Berne et al., 2010). Taken together, these findings shed light to a very important process taking place within microbial communities, where eDNA becomes a polyvalent molecule used for communication, feeding, genetic exchange and as a structural compound in biofilms formation. In addition, it should be stressed that all these functions are very relevant at the community level, and could represent an altruistic act of the subpopulation releasing eDNA to benefit kin cells. On the other hand, we should not forget that the studies on the function of the eDNA have been performed under laboratory conditions. In natural environments, microbial communities are usually composed of a mixture of different species (Earl et al., 2008; Kolter, 2010) and mechanisms such as predation or fratricide would gain more sense (Nakamura et al., 2008; Thomas et al., 2009). In any case, the studies done so far will certainly help to understand how these complex communities develop and interact in their natural environment.

## Author contributions

All authors contributed substantially to the writing of the manuscript. AI and JG designed the Figures.

### Conflict of interest statement

The authors declare that the research was conducted in the absence of any commercial or financial relationships that could be construed as a potential conflict of interest.

## Abbreviations

CSF: Competence and Sporulation Factor
CSP: Competence-Stimulating Peptide
eDNA: extracellular DNA
GGI: Gonococcal Genetic Island
HGT: Horizontal Gene Transfer
MV: membrane vesicles
PQS: Pseudomonas Quinolone Signal
QS: Quorum Sensing
T4SS: Type 4 Secretion System.
